# Mothers’ strategies for creating positive breastfeeding experiences: a critical incident study from Northern Sweden

**DOI:** 10.1186/s13006-022-00474-9

**Published:** 2022-05-08

**Authors:** Anna Jacobzon, Åsa Engström, Birgitta Lindberg, Silje Rysst Gustafsson

**Affiliations:** Department of Nursing Science and Medical Technology, Luleå University of Technology, Luleå, Sweden

**Keywords:** Breastfeeding, Attitude, Strategies, Breastfeeding support, Critical incident technique

## Abstract

**Background:**

Positive breastfeeding experiences positively influence subsequent attitudes towards breastfeeding, and increase mothers’ confidence, self-efficacy, motivation and intention to breastfeed. However, the strategies that mothers find useful and effective for creating positive breastfeeding experiences remain largely unknown. The aim of our study was thus to describe experience-based knowledge from mothers about strategies for creating positive breastfeeding experiences.

**Methods:**

The study followed a qualitative design involving the critical incident technique. Data were collected with an online survey containing open-ended questions that was administered to a Sweden-based parenting group on Facebook in September 2018. Ultimately, 340 incidents from 176 women were identified as offering strategies for creating positive breastfeeding experiences. Data from the written replies were extracted as textual units, condensed and categorised until categories were mutually exclusive, which resulted in six categories.

**Results:**

Participating women were on average 31.2 years old and the median number of children per participant was two. Mothers’ strategies for creating positive breastfeeding experiences generally included being calm and accepting that initiating breastfeeding takes time and can be difficult initially. Participants described feeling close to the baby by maintaining skin-to-skin contact and being present in the moment by taking time to appreciate the child and the breastfeeding situation, and temporarily forgetting about the world and simply being with the child in the here and now. Participants advocated baby-led breastfeeding and following correct techniques. They also described the importance of keeping an effortless mindset about breastfeeding to prevent perceiving breastfeeding as a compulsion. Mothers described acquiring knowledge about breastfeeding so that they could be prepared if breastfeeding problems occurred and getting support from professionals and family was described as significant for having a positive breastfeeding experience. Caring for oneself and one’s body, with aids if necessary, were described as important strategies, as were having a positive attitude and a strong desire to breastfeed.

**Conclusion:**

Because positive breastfeeding experiences and support are predictors of future breastfeeding initiation and duration, assisting women in creating positive breastfeeding experiences is important. Asking mothers to formulate strategies that they find useful could facilitate breastfeeding by making their approaches more conscious and visible.

## Background

Having a child is one of life’s greatest and most amazing experiences. However, the time after childbirth can nevertheless cause significant strain for new parents who may experience not only reduced sleep related to the child’s sleeping habits and crying patterns but also difficulties with breastfeeding [1, 2].

The World Health Organization (WHO) recommends exclusive breastfeeding for six months followed by breastfeeding with additional food until children are at least two years old [3]. In Sweden, the National Board of Health and Welfare recommends exclusive breastfeeding for six months followed by breastfeeding with additional food up to one year of age or for as long the infant and mother would like [4]. In Sweden, breastfeeding rates for children less than six months old decreased between 2004 to 2010, after which the downward curve levelled out. Breastfeeding rates in Sweden show that most women breastfeed exclusively for at least one week (75%) and half of these women continue to breastfeed exclusively for four months (50%). However, few women breastfeed exclusively for six months (13%) or continue to breastfeed for one year (27%) when almost every infant has received additional food [5].

Breastfeeding is an important predictor of health outcomes and has long-term health benefits for both women and their babies [6–10]. Health benefits of breastfeeding are important incentives to breastfeed and represent a set of underlying factors of society’s commitment to protect, promote and support breastfeeding. That commitment is manifest in the implementation of baby-friendly hospitals and the mission of many maternal healthcare services around the world to follow the WHO’s Ten Steps to Successful Breastfeeding [11]. Support for breastfeeding is a decisive factor for success and can consist of formal support (i.e., from healthcare professionals) and / or informal support (i.e., from partners, family members and friends) [12]. Meanwhile, encouragement to breastfeed and guidance in taking positive approaches towards breastfeeding from healthcare professionals are supporting factors. In fact, providing evidence-based breastfeeding support in sensitive, individualised ways through a mutual and respectful dialogue has been shown to increase the likelihood of positive breastfeeding experiences. In addition, being prepared by acquiring information during pregnancy both by written and oral information has been shown to increase the possibility of having positive breastfeeding experiences [13].

Breastfeeding experiences are important because positive experiences positively affect a mother’s future tendency to breastfeed [14]. In particular, such experiences increase the mothers’ subsequent attitude towards breastfeeding and their confidence, self-efficacy, motivation, and intention to breastfeed [15]. Positive breastfeeding experiences also promote high self-efficacy in breastfeeding as well as maternal self-efficacy during the transition to parenthood [12, 16]. At the same time, the decision to breastfeed is influenced not only by experiences of success with the practice [14] but also by the vicarious experiences of significant members in the mother’s social network, including family members and friends [12, 14]. Last, positive experiences with breastfeeding cultivate positive views on breastfeeding, which can also bolster positive attitudes towards the practice and the motivation to breastfeed [17]. It is therefore important that women have positive breastfeeding experiences and share them with others, which can contribute to shaping cultural norms and views on breastfeeding.

Although knowledge of mothers’ experiences enables healthcare providers to adapt care to respond to and meet the specific needs of each family [18], little is known about what strategies mothers find useful and effective for creating positive breastfeeding experiences. A reason may be that such experience-based knowledge is often implicit and thus requires qualitative methods to systematise it and make it visible. By systematically collecting and studying experience-based knowledge however, researchers can expand, enrich and improve the scientific basis for advice and care that promotes positive breastfeeding experiences. Given all of the above, the aim of our study was to describe experience-based knowledge from mothers about strategies for creating positive breastfeeding experiences.

## Methods

### Design

The study has a qualitative design conducted by following the critical incident technique (CIT). Focused on solving specific problems, CIT is a suitable method for research aimed at collecting and systematising experience-based knowledge [19]. The reporting of the study herein follows the COREQ (consolidated criteria for reporting qualitative research) Checklist [20].

### Participants

The selection of study participants was non-randomised, consisting of a convenience sample from a Facebook group for mothers in northern Sweden with approximately 5000 members. This group was selected because it is the largest Facebook group for mothers in Northern Sweden. The study participants were recruited through an announcement in the group. The announcement contained a link to a webpage describing the study’s aim and procedures, and the participants were informed that completing and submitting the survey on the page indicated their consent to participate in the study. Inclusion criteria were being a mother of at least one child, being more than 18 years old and having experience with breastfeeding.

### Data collection

Data were collected through open-ended questions in an online survey. The survey was launched in September 2018 and available for five weeks, during which reminders were published after 10 and 20 days. The survey first gathered the demographic information of the participants, including their age, gender, number of children and their ages, marital status, country of birth and employment.

Next, the survey contained three open-ended questions: “What strategies have you used to create a positive breastfeeding experience?”, “What advice about breastfeeding would you give a new parent?” and “Do you have anything you would like to add about breastfeeding?” The two first open-ended questions were carefully constructed in accordance with Flanagan’s [19] recommendations to ask questions that reflect the study’s general aim and to ask for a summary. The first question was worded to gather participants’ perspectives on the study’s focal topic (i.e., strategies for positive experiences in breastfeeding), whereas the second was worded to gather summaries of their experiences in brief, condensed statements. Last, the third question was asked to reveal additional information that might have been missed during the first two questions. Before the survey was posted, all three questions were pilot-tested with four mothers in order to evaluate the questions’ understandability and relevance, after which minor adjustments in the wording were made to increase the clarity of the questions.

### Data analysis

Using Flanagan’s method [19], we analysed the data following the CIT, which is considered to be suitable for studying human behaviour in specific situations. The method’s first step is developing a general frame of reference corresponding to the study’s aim that can promote the classification of *critical incidents*, defined as behaviours that are decisive for particular outcomes. In our study, critical incidents were mothers’ strategies for creating positive breastfeeding experiences. Two authors (AJ and SG) independently identified such incidents in the data and evaluated their combined findings until reaching consensus. Ultimately, 340 such incidents were identified. According to Flanagan [19], the number of critical incidents collected is more important than the number of participants and should total at least 100 to achieve data saturation.

The second step of the CIT, category formulation, generally involves the inductive development of a set of major categories and subcategories. Therein, critical incidents that are similar in content are organised and combined until all the incidents have been classified in final categories and the final categories are mutually exclusive. According to Flanagan [19], the categories might require modification or redefinition during the process of analysis, and a need for new categories might arise. In our study, all authors performed categorisation together, and the analysis process went back and forth until there were six final categories remaining.

The third step of the CIT is determining the most appropriate level of specificity and generality to adopt in reporting the data. Herein, due to the heterogeneity of incidents identified, the results of our study are presented as general behaviours in order to increase their coherence.

## Results

The participants (*n* = 176) were 31.2 years old on average (range: 21–47 years). All were women, and the median number of children per participant was two. Overall, 47.2% (*n* = 84) were married, 48.3% (*n* = 87) were cohabiting, and 3.9% (*n* = 7) were single. The vast majority (93.8%) was born in Sweden and gainfully employed (87.1%). From the responses of the participants, we identified 340 critical incidents offering strategies for creating positive breastfeeding experiences, which we grouped in six categories.

### Calm and closeness

Participants highlighted the importance of remaining calm, accepting that initiating breastfeeding takes time and not feeling stressed if breastfeeding is initially difficult. Other important factors were taking the time to become familiar with the child and learning both how to breastfeed and that breastfeeding becomes easier with time:

*“The baby is also new to breastfeeding, you can do it together.” Participant No. 3, 33 years, one child*

Participants also stressed the importance of breastfeeding in calm, comfortable places made as cosy as possible (e.g., on the sofa with pillows and blankets) and where they could cuddle with the child. They added that calm surroundings are necessary because breastfeeding may not always commence immediately, and that breastfeeding can be more difficult when the mother is stressed:

*“If you’re calm, then the baby is calm, and breastfeeding gets easier”. Participant No. 134, 35 years, one child*

Participants recommended actively bonding with their children by getting close and holding them skin-to-skin. They described how mother–child intimacy benefitted from their being present in the moment — that is, taking time to appreciate the child and the breastfeeding situation, temporarily forgetting about the world and simply being with the child in the here and now. Talking or singing in a soft voice, gently caressing the child’s back and making eye contact were also described as being significant when breastfeeding:

*“Make as much skin-to-skin contact as possible in the start. Cuddle with the baby under a blanket”. Participant No. 83, 32 years, one child*

### Practising baby-led feeding following correct techniques

To create positive breastfeeding experiences, participants underscored the significance of responsive breastfeeding ─that is, being sensitive to the child’s signals and initiating breastfeeding in response to the child’s cues. They stated that being perceptive about the child and initiating breastfeeding before children become too hungry make breastfeeding easier:

*“Be perceptive about the baby’s signals and breastfeed whenever the baby wants to”. Participant No. 51, 32 years, two children.*

Participants added that sitting comfortably makes it easier to hold the child in a correct position, which in turn helps the child to secure an appropriate grip when suckling. Some described that it was initially difficult to do so however, and reported testing different breastfeeding positions. Taking breaks and re-initiating breastfeeding were also mentioned as successful strategies:

*“In the beginning, it was difficult to get into a good position and to get the baby to have a correct grip. It was frustrating and stressful and often made me sad. But taking breaks and starting over helped me a lot”. Participant No. 10, 29 years, two children.*

### Lowering demands

The fear of being unable to breastfeed was reported to cause feelings of uncertainty that were liable to inhibit breastfeeding. In response, participants thought it was important to keep an effortless mindset about breastfeeding to prevent perceiving breastfeeding as a compulsion. Several participants stated that it was nice if breastfeeding worked, but if not, then it was not the end of the world, and the alternative of feeding the child formula was always available. They added that knowing that formula was an option and that their children would not starve made them relax, which in turn facilitated breastfeeding.

*“I let go of all thoughts about the ‘demands of breastfeeding’. From the start, that made me feel relaxed about breastfeeding, and I didn’t get stressed about it if it was sometimes complicated or didn’t work”. Participant No. 67, 30 years, two children.*

Participants additionally emphasised the importance of not paying too much attention to the demands of others present during breastfeeding, or what they might think. Instead, they advised focusing on what would be best for the mother and the child and that, if breastfeeding did not work, then they could feel content about having tried instead of feeling guilt or shame:

*“Don’t pay any attention to what people around you think. Focus on what’s best for you and your baby. If breastfeeding doesn’t work, then at least you tried. Don’t feel guilty. The most important thing is that you and your baby are well”. Participant No. 21, age unknown, two children.*

The participants also recommended ignoring the demands of housekeeping. They stated that breastfeeding requires focus and takes time, which can be better spent with their young children instead of chasing the idea of a perfect home:

*“Do only what’s necessary at home. It [the house] doesn’t need to be spotless”. Participant No. 34, 22 years, two children*

### Acquiring knowledge and support

Participants described becoming informed about breastfeeding as an important strategy for being prepared for and knowing what to expect from the activity. To that end, recommended strategies were reading about breastfeeding, taking a course, talking with friends and / or others and seeking information from professionals in parenting groups. Gaining knowledge about the biology of breastfeeding and what to do when difficulties occurred helped to create a positive breastfeeding experience:

*“For me, the foundation for a positive breastfeeding experience was established during pregnancy, when I took a course about breastfeeding that gave me knowledge about how breastfeeding works from a biological standpoint. That made me feel sure that ‘the body knows how to do this’ and that it [breastfeeding] wasn’t anything to worry about”. Participant No. 67, 30 years, two children.*

Participants also characterised both professional and social support as being pivotal for creating positive breastfeeding experiences. Advice and support from midwives in maternity wards or nurses at health clinics were considered especially helpful:

*“Take it easy and ask for help if it [breastfeeding] doesn’t work. There are professionals who you can ask”. Participant No. 77, 28 years, one child*

Support from partners, family members, friends and social internet forums was also described as valuable:

*“Every baby is unique, and breastfeeding can be hard in the beginning, so it’s okay to feel that you want to give up. But at that point it’s important to get support from people around you”. Participant No. 33, age unknown, six children.*

If they had received unhelpful and / or contradictory advice, then the participants described having a strategy to seek help elsewhere ─ for example, from specialised breastfeeding clinics or online support groups for mothers to get the help they needed:

*“I seem to remember getting an overwhelming amount of information and advice about breastfeeding to prevent the baby from getting a stomach ache: everything from pumping out the first milk to avoiding all sorts of different foods and even breastfeeding lying down. The support group for mothers was a great help that allowed me to take stock of everyone’s different experiences with breastfeeding and to realise that what works varies from person to person”. Participant No. 160, 35 years, two children.*

### Practising self-care and using aids

Participants additionally stressed the importance of maintaining physical well-being. This included keeping the breasts warm to prevent breast engorgement, and eating and drinking well while breastfeeding, in order to manage breastfeeding. Self-care implied caring for oneself and being kind to oneself, as well as treating their body with respect and gratitude. Another strategy was wearing comfortable clothes that could facilitate breastfeeding. To avoid sore nipples, pain and breast engorgement, some participants advocated using aids such as nipple shields and, if necessary, breast pumps.

*“Wear clothes that make breastfeeding easy, including special breastfeeding linen and real bras. Remember to keep your breasts warm. Breast engorgement is NOT fun”. Participant No. 100, 26 years, one child.*

### Having a positive view and a strong desire to breastfeed

Participants stated that creating positive breastfeeding experiences benefitted from having positive expectations and a strong desire to breastfeed. Their advice was to focus on the positive aspects of breastfeeding despite its obvious challenges. Some mothers also highlighted the importance of not over-analysing the breastfeeding situation. At the same time, they reported not really having any strategies, for they did not see any alternative to breastfeeding. In any case, the participants also described the benefits of breastfeeding, even when they felt tired, and recommended other mothers to be persistent and to not give up:

*“Try to have a positive view about breastfeeding. When it works, it’s the best and cosiest thing that there is”. Participant No. 88, 39 years, one child*

Participants advocated having a positive view that their bodies know what to do during breastfeeding – in short, to trust and follow their instincts. That strategy included having a positive view about their bodies, in the fact that breastfeeding would work and that their milk would be sufficient for their children:

*“When I followed my instincts, I felt so much better, and breastfeeding worked better, too”. Participant No. 62, 32 years, two children*

## Discussion

The major finding of our study was that mothers’ strategies for creating positive breastfeeding experiences generally included being calm and close to the child and being present in the breastfeeding moment. Lowering demands was also described as an important factor for positive breastfeeding experiences. To facilitate breastfeeding, mothers additionally recommended baby-led breastfeeding with correct techniques, and they suggested reading about breastfeeding in order to acquire knowledge about the activity and to ask for support if needed. Taking care of oneself and one’s body, using aids if necessary, as well as having a positive view and a strong desire to breastfeed, were also described as important factors of positive breastfeeding experiences.

Above all, participating mothers described that being calm and in closeness with their children was pivotal for creating positive breastfeeding experiences. Strategies to that end included maintaining skin-to-skin contact, making eye contact, caressing the child and talking to the child in a soft voice. Skin-to-skin contact and touch increase oxytocin secretion, which alleviates stress and pain, contributes to a sense of well-being and calmness, is linked to increased social interaction and, in the context of breastfeeding, induces milk ejection [21].

Such mother–child interaction during breastfeeding is important for establishing eating habits and attachment, and, indeed, physical presence and eye contact are central to what is called *responsive feeding*.

According to Silva, Costa and Giugliani [22], responsive breastfeeding involves being sensitive to the child’s signals and feeding slowly and patiently according to the child’s cues. This is because breastfeeding is an opportunity to learn from, love and talk to children, maintaining eye contact contributes to positive breastfeeding experiences that encourage children to eat without stress or distractions [22]. Eye contact and gazing are non-verbal cues that play a central role in communication; for example, an infant’s gaze can indicate hunger, hence the need for food, or satiation. Sensitivity to a child’s signals promotes responsive feeding and is essential to baby-led feeding [23]. The mothers in our study additionally highlighted that because infants are also new to breastfeeding, succeeding with breastfeeding was a team effort. That notion aligns with the concept described by Burns, Fenwick, Sheehan and Schmied that breastfeeding is a relationship between the mother and child [24].

To foster positive breastfeeding experiences, the mothers also emphasised the importance of lowering demands. Such demands could relate to housekeeping, for example, which the mothers advised taking a relaxed attitude towards while regularly engaging in breastfeeding. The mothers also highlighted the importance of keeping an effortless mindset about breastfeeding to avoid feeling a pressure to breastfeed. The mothers reported that fear of being unable to breastfeed could cause feelings of uncertainty that were liable to inhibit breastfeeding and by lowering demands that breastfeeding “must work” they felt more relaxed which in turn had a positive effect on breastfeeding. Personal, cultural and ideological views on feeding methods influence not only breastfeeding experiences but also perceptions of what constitutes so-called “good motherhood”.

Motherhood and breastfeeding are intimately intertwined such that performance and experiences in one will profoundly affect performance and experiences in the other [25]. In past work, Larsen et al. found that when mothers experienced breastfeeding as a duty and a personal responsibility, their confidence in their abilities as mothers was affected when problems with breastfeeding arose [26]. The expectation that breastfeeding is a natural process becomes replaced with the experience that breastfeeding is difficult and giving up on breastfeeding is often described by mothers as an experience of mixed feelings of failure and guilt [26]. Feelings of inadequacy can contribute to negative experiences with breastfeeding, which can consequently influence future breastfeeding initiation and duration.

Against that trend, Thomson, Ebisch-Burton and Flacking have posited that devising personal definitions of “good motherhood” can prevent feelings of failure, shame and judgement when breastfeeding fails [27]. For their part, as Cato, Sylvén, Henriksson and Rubertsson have argued, healthcare professionals should adopt more flexible stances on exclusive breastfeeding in order to not only reduce pressure to breastfeed among mothers who choose formula feeding but also reduce feelings of shame and guilt among ones who cannot or do not want to breastfeed [28]. Overall, participating mothers in our study expressed tremendous compassion towards other mothers, whether or not they had ever breastfed their children. By extension, participants stressed the importance of not feeling guilty if breastfeeding failed and of knowing, that giving up on breastfeeding does not mean that one is a bad mother.

The mothers in our study described having knowledge about breastfeeding as being important for having positive breastfeeding experiences. They reported that acquiring knowledge about breastfeeding techniques, what to expect of breastfeeding and what to do when breastfeeding-related problems occurred from not only reading and maternity education but also from discussions with midwives, nurses, partners and other mothers was helpful. The mothers also highlighted the importance of having support from midwifes, nurses, partners and family when initiating breastfeeding and to seek support elsewhere if receiving unhelpful and contradictory advice. It is well documented that support, in particular from trained professionals, can increase both duration and exclusivity of breastfeeding [29]. Several studies and meta-analysis have shown that women do not seem to get the support they need from healthcare professionals [30, 31]. Women with breastfeeding-related problems not finding skilled breastfeeding support are most likely to stop breastfeeding sooner than they had desired [32, 33].

Despite the importance of breastfeeding support, breastfeeding care is often fragmented leaving women with inadequate support [34, 35]. With increasingly short postnatal stays, the continuity of care regarding breastfeeding support needs to be improved and available for mothers to increase breastfeeding rates [36]. To the same end, it is essential for women to feel supported in creating positive breastfeeding experiences as a means to promote their motivation and intention to breastfeed in the future [15]. For healthcare professionals, providing individualised breastfeeding support can be a complex task, although following scientific evidence and the WHO’s recommendations should be viewed as a minimum.

The women in the study conducted by Blixt et al. wanted healthcare professionals to describe all options before advising which to follow, because options enable women to make informed decisions that align with their feelings and desires [13]. Although parents predominantly agree that breastfeeding is the best way to feed infants they desire and expect that they will be offered factual information related to their personal infant feeding choices, provided in a sensitive and non-judgemental manner [31]. At the same time, providing options can cause dilemmas for the primary healthcare nurse or midwife because by doing so the effort to promote exclusive breastfeeding and breastfeeding duration as recommended by the WHO [3] can be undermined, especially because breastfeeding is an important predictor of health for both mothers and children [6, 8, 10].

In fact, our study revealed that some mothers who perceived formula as an acceptable alternative to breastfeeding experienced reduced stress and pressure, which increased their likelihood of having positive breastfeeding experiences. After all, some women may fear that their breastmilk is insufficient nutrition and that their children may starve, largely because ensuring that children are not hungry is deeply ingrained in humans and can cause stress for mothers even when problems do not arise in feeding. As an antidote, supplementation with formula can heighten their sense of control over their children’s nutritional intake and assure them that their children are not hungry or starving.

In contrast to the other strategies, the mothers in our study had to create positive breastfeeding experiences; furthermore, the strategy to introduce formula as an alternative to increase the possibility of having a positive breastfeeding experience also poses some negative implications. Introducing formula at any age before 12 months is strongly, negatively associated with breastfeeding at 12 months [37]. Among other negative consequences of keeping formula as an option, in-hospital formula supplementation without medical cause and combining breastfeeding with formula both reduce the frequency and duration of breastfeeding [38, 39]. Thus, although reduced pressure to breastfeed can be perceived to promote positive breastfeeding experiences, introducing formula can reduce breastfeeding and therefore be counter-productive to the WHO’s recommendations [3]. Beyond that, in their study, Hvatum and Glavin [25] found that encouraging breastfeeding can also become or be misconstrued as pressure to breastfeed. Indeed, some mothers in their sample described feeling as though they were breaking the law if they could not or did not want to breastfeed. Likewise, Larsen et al. [26] have described how support for breastfeeding can become so focused on breastfeeding as the only correct method “that it actually works counter to the good intentions about supporting mothers to breastfeed”.

For an alternative source of encouragement, the mothers in our study stressed the importance of having a positive view and a strong desire to breastfeed. Support from healthcare professionals can influence women’s personal confidence in breastfeeding. In a metasynthesis by Schmied et al. [40], the result showed that if the intended support was perceived as supportive, women felt they were listened to and given realistic information, which increased confidence and sense of control. Those findings corroborate Blixt et al.’s [13] advice for healthcare professionals to provide breastfeeding support to women that is sensitive and individualised enough to strengthen their self-confidence, including by enabling individual decision-making and both supporting and respecting their breastfeeding goals and decisions.

In that light, the notion that breastfeeding is not only a natural process but also a competency that can be learned is central to the dynamic view on breastfeeding as a set of ability that can be developed and strengthened with the right help and appropriate knowledge [24]. That dynamic view on breastfeeding is also characterised by a less categorical approach in which breastfeeding is not only “exclusive”, “in part” or “not at all”. Instead, breastfeeding could be seen as operating on a scale from no breastfeeding to exclusive breastfeeding. Knowledge of the strategies that women themselves propose to create positive breastfeeding experiences may be important to provide individually tailored support and thus enable the mother to reach the higher end of the breastfeeding scale, if that is her goal. Asking mothers to formulate what strategies they find helpful could also be a part of facilitating more conscious and visible approaches to breastfeeding.

### Strengths and limitations

This descriptive study, designed to capture the strategies that mothers use to generate positive breastfeeding experiences, has limitations. For one, although generalisation is not typically the principal objective of qualitative studies, the non-probability convenience sample of the qualitative methodology limits the generalisability of the study’s results. For another, even though 176 mothers answered the survey, and 340 critical incidents were identified, the response rate was low given that the group had approximately 5000 members at the time of the survey. It also remains uncertain whether all members were active and in fact saw the announcement posted in the group or why some group members did not want to participate. Although we do not know whether mothers who chose not to participate would have proposed different strategies, the number of critical incidents that we collected indicates that data saturation was achieved. Among other limitations, data in our study were collected with an online survey, because we wanted strategies from a broad group of mothers with different backgrounds and experiences. Whereas Flanagan [19] has claimed that using questionnaires to collect data is suitable in large samples and that data for the CIT collected via questionnaires do not essentially differ from data acquired via interviews, using interviews could also have raised the risk of social desirability bias, because motherhood and breastfeeding are associated with stigma if the strategies used are not considered to be socially acceptable.

Data extraction was performed by two authors independently, and extracted data were compared and discussed until consensus was reached. The process of analysis was performed by all authors and critically reviewed and discussed in various steps to ensure triangulation and peer scrutiny. Final categories are illustrated with participants’ quotations to illustrate the analysis of data. The data collected was concisely worded and required a low level of interpretation. Participants did not have any established relationship with any of the researchers, and member checks were not performed, because all data were collected anonymously. Iterative questioning was used by requesting summaries, and the data collected indicated internal consistency. The study’s findings are in agreement with literature in the field and are reported in accordance with COREQ guidelines [20] to ensure the description of the context and methods of the study.

## Conclusion

Considering that positive breastfeeding experiences and support are predictors of future breastfeeding initiation and duration, it is important to assist mothers in creating positive breastfeeding experiences. When breastfeeding is perceived as voluntary, stress is relieved, thereby making the breastfeeding experience less demanding and more pleasurable. Striving to comply with WHO’s recommendations [3] to breastfeed exclusively for six months and breastfeeding with additional food until the child is at least two years is important and a benchmark that all healthcare professionals should seek to attain. However, it is also important that breastfeeding is promoted in a sensitive, non-judgemental manner knowing that giving up on breastfeeding often is described by mothers as an experience of mixed feelings of failure and guilt [26]. In that light, participating mothers in our study expressed tremendous compassion towards other mothers, whether or not they had ever breastfed their children. By extension, participants stressed the importance of not feeling guilty if breastfeeding failed and that giving up on breastfeeding does not mean that one is a bad mother. Knowledge of the strategies that women have proposed to create positive breastfeeding experiences may be important to provide individually tailored support and thus enable mothers to reach the higher end of the breastfeeding scale, if that is their goal. Asking mothers to formulate what strategies they find helpful could also be a part of facilitating more conscious, visible approaches to breastfeeding.

## Data Availability

The datasets used and / or analysed during the study are available from the corresponding author upon reasonable request.

## Abbreviations

CIT: Critical incident technique
COREQ: Consolidated criteria for reporting qualitative research
WHO: World Health Organization
